# The True Relations of Filth to Diphtheria

**Published:** 1889-05

**Authors:** A. Jacobi


### THE TRUE RELATIONS OF FILTH TO DIPHTHERIA.

"Diphtheria is a contagious disease. There is probably no spontaneous origin of diphtheria any more than there is a spontaneous origin of cholera or scarlatina. When an attack of diphtheria has made its appearance, it is well enough to examine the hygienic condition of the house, *with its deteriorating influences on the general health of the inmates, and look after the source of the case* in the persons of friends, attendants, and help."

In my "Remarks on the Nature and Treatment of Diphtheria," made by invitation before the Section of Diseases of Children of the British Medical Association, August, 1888 (*British Medical Journal*, September 22, 1888), there are found the following sentences: "*Foul air and sewer gas do not create diphtheria;* they do create dysentery and typhoid, or such a condition of general ill-health and malaise as to afford the diphtheritic virus a ready resting-place. There were plenty of malodorous privies and foul smells fifty years ago, but no epidemic of diphtheria. Besides, and mainly through the careful observations of English physicians, such as are contained in Dr. George Turner's report on diphtheria in lower animals and many others, the sources from which diphtheria may come are very many. Pigeons, fowls, turkeys, chickens, pheasants, cats, horses, sheep, cows, are just so many sources of diphtheria for man. Foods of all kinds, vegetables, and milk will transmit it. It sticks to furniture, floors, and wall paper, railroad cushions and school desks. No spontaneous generation is required to explain its ravages."—*A. Jacobi, M. D., Archives of Pediatrics.*

